# Identifying global expression patterns and key regulators in epithelial to mesenchymal transition through multi-study integration

**DOI:** 10.1186/s12885-017-3413-3

**Published:** 2017-06-26

**Authors:** Princy Parsana, Sarah R. Amend, James Hernandez, Kenneth J. Pienta, Alexis Battle

**Affiliations:** 1 Department of Computer Science, Johns Hopkins University, Baltimore, MD 21218 USA; 2 Department of Urology, Johns Hopkins University School of Medicine, Baltimore, MD 21287 USA

**Keywords:** EMT, Metastasis, Prostate cancer, C1orf116, Multi-study integration

## Abstract

**Background:**

Epithelial to mesenchymal transition (EMT) is the process by which stationary epithelial cells transdifferentiate to mesenchymal cells with increased motility. EMT is integral in early stages of development and wound healing. Studies have shown that EMT could be a critical early event in tumor metastasis that is involved in acquisition of migratory and invasive properties in multiple carcinomas.

**Methods:**

In this study, we used 15 published gene expression microarray datasets from Gene Expression Omnibus (GEO) that represent 12 cell lines from 6 cancer types across 95 observations (45 unique samples and 50 replicates) with different modes of induction of EMT or the reverse transition, mesenchymal to epithelial transition (MET). We integrated multiple gene expression datasets while considering study differences, batch effects, and noise in gene expression measurements. A universal differential EMT gene list was obtained by normalizing and correcting the data using four approaches, computing differential expression from each, and identifying a consensus ranking. We confirmed our discovery of novel EMT genes at mRNA and protein levels in an in vitro EMT model of prostate cancer – PC3 epi, EMT and Taxol resistant cell lines. We validate our discovery of *C1orf116* as a novel EMT regulator by siRNA knockdown of *C1orf116* in PC3 epithelial cells.

**Results:**

Among differentially expressed genes, we found known epithelial and mesenchymal marker genes such as *CDH1* and *ZEB1*. Additionally, we discovered genes known in a subset of carcinomas that were unknown in prostate cancer. This included epithelial specific *LSR* and *S100A14* and mesenchymal specific *DPYSL3*. Furthermore, we also discovered novel EMT genes including a poorly-characterized gene *C1orf116*. We show that decreased expression of *C1orf116* is associated with poor prognosis in lung and prostate cancer patients. We demonstrate that knockdown of *C1orf116* expression induced expression of mesenchymal genes in epithelial prostate cancer cell line PC3-epi cells, suggesting it as a candidate driver of the epithelial phenotype.

**Conclusions:**

This comprehensive approach of statistical analysis and functional validation identified global expression patterns in EMT and candidate regulatory genes, thereby both extending current knowledge and identifying novel drivers of EMT.

**Electronic supplementary material:**

The online version of this article (doi:10.1186/s12885-017-3413-3) contains supplementary material, which is available to authorized users.

## Background

Cancer is the second leading cause of death in United States. Metastasis is the leading cause of cancer-related morbidity and mortality [1], but identifying tumors with metastatic potential remains a challenge [2]. Tumor metastasis is a multi-step process in which primary tumor cells disseminate from their site of origin to seed secondary tumors at a distant site [3]. It is believed that in a critical early event in cancer progression, metastatic cancer cells undergo an epithelial to mesenchymal transition (EMT). During EMT, stationary epithelial cells lose cell polarity and transdifferentiate to spindle-shaped motile mesenchymal cells. EMT is a crucial physiologic process involved in early development during embryogenesis and organogenesis. It also plays an important role in tissue regeneration and wound healing. However, in cancer EMT may contribute to tumor progression and malignant transformation. Several epithelial cancer cells have been described to undergo EMT transform to a more malignant phenotype [4] that can further promote formation of secondary tumors [5].

**Fig. 1:**
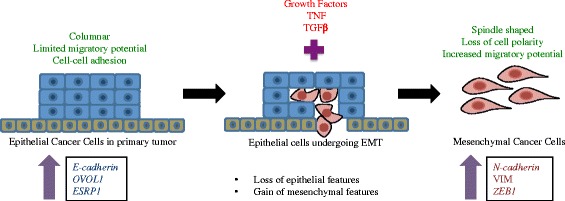
Epithelial to Mesenchymal Transition. During EMT, non-motile epithelial cells trans differentiate to mesenchymal cells with increased migratory potential. During this, cells show decreased expression epithelial specific genes that include *E-cadherin, OVOL1* and *ESRP1*. At the same time, expression of mesenchymal genes such as *N-cadherin, VIM* and *ZEB1* increases

The role of EMT has been frequently debated in clinical cancer metastasis [6]. However, several in vitro studies have shown that epithelial cancer cells can undergo EMT in response to a combination of signals from the tumor microenvironment [2]. During EMT, cells go through multiple morphological and biochemical changes resulting in loss of epithelial properties coupled with gain of mesenchymal characteristics [7–21]. Microarrays have been widely used to study gene expression patterns of cell populations under different experimental settings, including EMT-inducing conditions (Fig. 1). While there have been many studies investigating the effect of a gene or pathway in EMT, none have explored the universal changes across multiple cancer tissue types or EMT induction methods.

Several gene expression datasets examining EMT in a variety of different cell lines under different conditions are available on open access databases such as Gene Expression Omnibus (GEO) [22]. It has been demonstrated that re-use and aggregation of public gene expression data facilitates discovery of signals too weak to be detected in an individual experiment [23–26]. Gröger et al. performed meta-analysis of 18 EMT gene expression studies and identified 130 core-EMT genes, which were differentially expressed in at least 10 of the 18 studies [27]. Genes such as *TGFB*, *GNG11*, *TIMP1*, *ETS1*, *S100A14*, *DPYSL3* and *C1orf116* that we discovered as differential EMT, were not found in their core EMT gene list. Furthermore, we experimentally validated some of these genes (*S100A14, DPYSL3* and *C1orf116*) in PC3 epithelial, PC3-EMT and PC3-taxol resistant cell lines confirming their association in EMT. Also, each dataset in [27] was confined by small sample size per class (n < =6). The drawback with underpowered studies are: a) low probability of identifying true effects b) overestimation of effect size [28, 29]. Therefore, genes that showed consistent moderate effects across datasets could be missed. In contrast, systematic integration of multiple studies promotes reliable detection of consistent gene expression changes that may otherwise be false negatives in results obtained from individual experiment [30]. At the same time, it helps avoid false discoveries that could result from intra-study variability resulting from single experiment.

Batch effects and noise introduce spurious signal and correlations in microarray gene expression data [31, 32]. Therefore, data normalization is crucial in order to correct the data for unwanted biological or non-biological effects. However, Groger et al. do not account for batch effects, cross-platform differences, or cross-tissue effects in their meta-analyses study that could potentially lead to false positive findings.

**Fig. 2:**
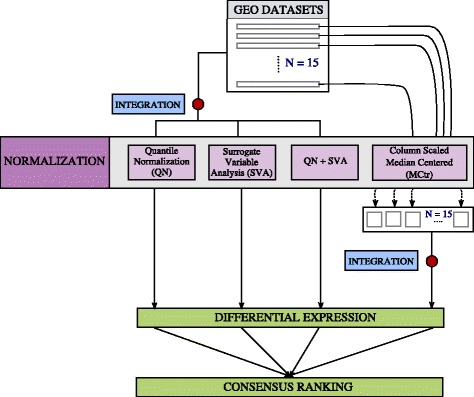
Workflow for multi-study data integration, normalization and identification of candidate universal EMT genes

In this study, to identify universal EMT genes common across multiple cancer types, we integrated 15 independent gene expression studies representing 12 cell lines (49 epithelial and 46 mesenchymal phenotypes) from 6 cancer tissue types and multiple EMT induction modalities (Table 1, Additional file 1: Table S1). After correcting data to account for cross-study differences, cross-platform differences, and other sources of noise, we performed differential expression analysis and identified global changes in gene expression patterns between epithelial and mesenchymal states (Fig. 2). Importantly, our candidate gene list was enriched for EMT-related genes and we identified known markers of EMT. In addition, we also identified EMT genes that had only been described in a sub-set of malignant disease states, but were previously unknown in prostate cancer (e.g. *LSR*, *S11A14*, *DPYSL3*), implying a common EMT program across multiple cancer types. We further identified genes that had not been previously characterized in EMT in any disease state including *C1orf116*, which we then experimentally validated using siRNA knockdown in PC3 epithelial cells. This approach of multi-study integration enabled identification of differential EMT genes universal across different types of cancer. Functional validations of these genes indicate manifestation of molecular mechanisms contributing to EMT shared across disease types. This study also identifies an uncharacterized candidate novel EMT regulator gene *C1orf116*. These findings thereby extend our knowledge and understanding of EMT biology.

**Table 1:** Dataset information

GEO ID	Platform ID	Disease Type	Cell line	Samples*	Ref
GSE12811	GPL7319	Breast	MCF10A	3	[7]
GSE13915	GPL7785	Breast	BT549, EFM19	4	[8]
GSE18070	GPL570	Breast	MCF10CA1h	9	[9]
GSE28569	GPL6480	Breast	MCF10A	8	[10]
GSE39356	GPL6480	Breast	MCF-7	4	[11]
GSE8240	GPL3921	Breast	MCF10A	11	[12]
GSE12203	GPL2700	Colon	Caco-2	4	[13]
GSE14773	GPL570	Colon	HT29, SW480	8	[14]
GSE27424	GPL570	Esophageal	EPc2-hTERT	12	[15]
GSE26391	GPL6244	Liver	HCC-1.1, HCC-1.2	8	[16]
GSE14405	GPL570	Prostate	PC3, TEM4, TEM2	6	[17]
GSE22010	GPL6244	Prostate	PrEC-hTERT	2	[18]
GSE22764	GPL6884	Prostate	PC3	6	[19]
GSE43489	GPL570	Prostate	PC3	4	[20]
GSE12548	GPL570	Retinal pigment	ARPE19	6	[21]

*Indicates the number of samples included in our study

## Methods

### Data overview

We used 15 published EMT microarray gene expression datasets from GEO (Gene Expression Omnibus) (Table 1, Additional file 1: Table S1). This comprises of 95 observations (45 unique samples and 50 replicates), 49 epithelial and 46 mesenchymal cell lines exposed to different treatment modalities. The cell lines come from 6 different tissue types including breast, prostate, colon, esophageal, liver and retinal pigment and 4 different microarray platforms (8 chips), Affymetrix, Agilent, Stanford Microarray Database (SMD) and Illumina. All the datasets were downloaded in the format they were submitted to GEO. We mapped platform specific probe IDs to Ensembl IDs and gene symbols. When multiple probes mapped to same gene, we used median values to represent expression of that gene. We used 7276 genes common across all datasets.

### Data normalization

This work combined data from multiple studies spanning diverse cell lines and different platforms. Batch effects and noise are inherent in gene expression data. To account for confounders in data as a result of cross-study and cross-platform effects, we used multiple correction methods, such as quantile normalization (QN), Surrogate Variable Analysis (SVA), Quantile normalization followed by SVA and Column Standardized Median Centered (MCtr). We merged all 15 datasets into one matrix prior to quantile normalization and SVA. For CMSC, we individually processed each study and combined them after normalization.

#### Quantile normalization

Quantile normalization makes the gene expression distribution of each sample in the dataset the same. Given a dataset D, with ‘g’ genes and ‘n’ samples:

- Sorts each column in D
- Computes mean for each row and assigns it to each element in the row giving D’
- Finally, it rearranges columns in D’ such that it has the same ordering as original D, thus giving normalized data, D_normalized

At the end of this, each column in D has the same distribution [33].

#### Surrogate variable analysis

Surrogate variable analysis allows us to preserve the phenotype signal of interest (epithelial and mesenchymal). It estimates known and hidden confounding factors using Singular Value Decomposition on residual variation matrix. We regress out estimated surrogate variables from gene expression data to get SVA normalized gene expression [34].

We also quantile normalize combined data followed by SVA to correct for hidden confounders.

#### Column standardized median centered

Samples from each study are standardized and median centered by gene as described in [35] and combined them.

### Differential expression analyses and concordance between normalization methods

With each of the normalized dataset, we used a two-sample t-test to identify differentially expressed genes between epithelial and mesenchymal states. Assuming equal variance, we compared the mean expression of a gene between the two populations. For each gene, we tested:

$$ \mathrm{Null}\ \mathrm{Hypothesis}:\kern0.5em {\mu}_{epi}={\mu}_{mes} $$

$$ \mathrm{Alternative}:\kern0.5em {\mu}_{epi}\ne {\mu}_{mes} $$

We ranked genes by raw *p*-values. We applied Bonferroni correction for multiple hypothesis testing.

To test concordance between normalization methods, we used spearman rank correlation to test association between gene ranks (*n* = 7276) obtained by different correction methods.

Assuming equal probability of error for each normalization method, we computed average rank for each gene across the four methods that represented the consensus position of each gene according to the differential expression test statistic (Fig. 2).

### Cluster evaluation of normalized data

To evaluate if normalization improved overall grouping of epithelial and mesenchymal phenotypes together, we clustered each of the normalized data using hierarchical clustering (with all 7276 genes). Next, to evaluate grouping we used Baker Hubert Index for cluster evaluation. Baker Hubert’s Index (BH) [36] is an adaptation of Goodman and Kruskal gamma statistic in the context of clustering.

$$ BH=\frac{S^{+}-{S}^{-}}{S^{+}+{S}^{-}} $$

Here, *S* ^+^ is the number of concordant quadraples and *S* ^−^ is the number of disconcordant quadraples. To compute BH, it tests all possible quadraples in the input.

Suppose we were testing quadruple samples *a* , *b* , *c* , *d*. And *d*(*a*, *b*) is the distance between samples *a* and *b*. A quadruple is concordant if it fulfills one of the following two conditions:

- *d*(*a*, *b*) > *d*(*c*, *d*); And c and d are in same cluster and a and b are in different clusters
- *d*(*a*, *b*) < *d*(*c*, *d*); And a and b are in same cluster and c and d are in different clusters

A quadruple is disconcordant if:

- *d*(*a*, *b*) > *d*(*c*, *d*); And a and b are in same cluster and c and d are in different clusters
- *d*(*a*, *b*) < *d*(*c*, *d*); And c and d are in same cluster and a and b are in different clusters

Since we were interested in improvement in grouping of epithelial and mesenchymal samples, we used phenotype vector as cluster assignment for evaluation.

### Gene co-expression module detection using WGCNA

With 200 DE genes from QN + SVA data, unsigned co-expression network was constructed using the WGCNA package in R [37]. Since we used differentially expressed genes, prior to constructing networks, the effect of phenotype (epithelial and mesenchymal) from each gene was removed using a linear model.

$$ {E}_i=\mu +{\beta}_1\cdotp {P}_i+\epsilon $$

where, *μ* is the mean effect, *E*_*i*_ is the expression of a gene in sample *i, β*_1_ is the regression coefficient of phenotype, *P*_*i*_ is the phenotype label for sample *i* and *ϵ* ~ *N*(0, 1). Expression of gene $\widehat{E}$ after regressing out effect of phenotype is given by:

$$ \widehat{E_i}={E}_i-\left(\mu +{\beta}_1\cdotp {P}_i\right) $$

Next using this, we computed an adjacency matrix *a*_*ij*_ using pearson correlation:

$$ {a}_{i j}={\left| corr\left({e}_i,{e}_j\right)\right|}^{\beta} $$

where *e*_*n*_is the expression of gene *n* and *β* is the soft-thresholding power for weighted networks. Best scale-free topology fitting index *R* ^2^ was obtained at *β* = 5.5 (*R* ^2^ = 0.77). The adjacency matrix was then transformed to a topological overlap based similarity matrix given by:

$$ TO{M}_{ij}=\sum_u\frac{\sum_k{a}_{ik}{a}_{k j}+{a}_{ij}}{\mathit{\min}\left\{{\sum}_k{a}_{ik},{\sum}_k{a}_{jk}\right\}+1-{a}_{ij}} $$

The topological overlap between two nodes is the measure of relative interconnectedness. The TOM was then transformed to dissimilarity matrix:

$$ dissTO{M}_{ij}=1- TO{M}_{ij} $$

Genes were then clustered using average linkage hierarchical clustering.

Co-expression modules were derived from clustering dendrogram using Dynamic Tree Cut with hybrid method. This helped overcome the need for manually selecting a cut-off height. We set minimum module size to 15 since we were looking for modules among 200 genes. The expression profile of each module is represented by its eigengene, which is the first principal component of the module.

### RT-qPCR

RNA was isolated from cells at ~80% confluency using RNeasy kit (Qiagen) and subsequent cDNA libraries were prepared using Bio-Rad cDNA synthesis kit. TaqMan gene expression assays were used to determine mRNA expression levels using the following probes: β-actin Hs_1060665_g1, LSR Hs01076319_g1, S100A14 Hs04189107, DPYSL3 Hs00181665_m1, *C1orf116* Hs00539900_g1, OVOL1 Hs00970334, CDH1 Hs01023894, CDH2 Hs00983056_m1, ZEB1 Hs00232783_m1.

*Relative Expression Calculations*: In the qPCR, the target of interest in each sample is measured using at least three biological replicates. The Ct value for each biological replicate is calculated as an average of three technical replicates. Then the Ct value of each biological replicate is normalized to β-actin by subtracting it from the corresponding Ct value of β-actin (−ΔCt). The two groups of interest are compared using a Student’s t-test. The values plotted in the graph are the average of the base 2 anti-log transformations of -ΔCt for the biological replicates of interest divided by the average of the base 2 anti-log of -ΔCt for the reference group. The standard errors of the mean are determined from biological replicates.

### Western blot

Protein extracts were prepared using Frackleton-lysis buffer with protease inhibitors (Thermo Scientific 78,410), and samples were electrophoresed on 4–15% SDS-PAGE (Bio-Rad), transferred to a nitrocellulose membrane and blocked with casein blocking buffer (Sigma B6429). The list of antibodies used for western blotting is in Additional file 2: Table S6. The Licor Odyssey fluorescence scanner was used for visualizing the westerns.

### siRNA knockdown of *C1orf116*

C1orf116 siRNA (ThermoFisher, cat#: 4,392,420) with RNAiMAX transfection reagent (ThermoFisher) was used for siRNA transfections. Some alterations were made to manufacturer’s recommended protocol. Cells were seeded at a density result in 50% confluency the following day. Using a 6 well plate, 9 ul of RNAiMAX reagent and 3 ul (30 pmol) of siRNA (each diluted in 150 ul of Opti-MEM media) was added to each well the day after seeding. 72 h later RNA was isolated (Qiagen, Rneasy mini kit) from plates and gene expression was analyzed.

### *C1orf116* expression in cancer patient data

We identified publicly available published cancer patient (breast, prostate, esophageal, liver, colorectal, and lung) gene expression studies with at least 150 patients on Oncomine [38]. Gene expression data for studies (GSE17536 [39], GSE11121 [40], GSE25066 [41], GSE22358 [42], GSE7390 [43], GSE68465 [44], GSE31210 [45], and GSE21034 [46]) available on GEO were obtained using the GEOquery R package [47]. Probeset IDs corresponding to *C1orf116* were used. Gene level expression was obtained by aggregating multiple probe expression values with median. Wilcoxon rank sum test was used to test association between expression of *C1orf116* and grade, smoking status and cancer sample site. We also looked at association between tumor grade and *C1orf116* expression in 4 breast cancer, 1 colorectal cancer and 1 lung cancer studies from Oncomine. We adjust Wilcoxon rank sum *p*-values with bonferroni correction for a total of 23 tests performed for clinical associations (Table 2, Additional file 3: Table S7 and Additional file 4: Figure S7).

**Table 2:** Association of C1orf116 expression in lung and prostate cancer patients

Test group	Wilcoxon rank sum *p*-value	Bonferroni adjusted *p*-value
Lung cancer (Director’s Lung Challenge): grade [44]		
Grade1 vs Grade 2	1.4191e-06	3.27E-05
Grade 2 vs Grade 3	1.1481e-10	2.65E-09
Grade 1 vs Grade 3	2.6121e-17	6.00E-16
Lung cancer (Director’s Lung Challenge): Smoking Status [44]		
Never vs Past	0.006	1.38E-01
Past vs Current	0.006	1.38E-01
Never vs Current	0.0002	4.60E-03
Lung cancer (Okayama): Smoking status [45]		
Never smoker vs ever smoker	0.0586	1E + 00
Prostate cancer (Taylor): Tumor type [46]		
Primary vs Metastatic	0.0340	7.82E-01

## Results

We identified publically available gene expression microarray datasets that queried gene expression of cell lines induced to undergo EMT [7–21]. We confirmed the phenotype of the samples by referring to associated publications for immunohistochemistry staining and/or protein expression of known epithelial or mesenchymal markers (Table 1, Additional file 2: Table S1). 95 cell line observations (45 unique samples and 50 replicates) from 15 datasets that showed sufficient evidence of correct phenotypic labeling included 49 cell lines of epithelial phenotype and 46 cell lines of mesenchymal phenotype.

### Normalization methods show consistency in signal

**Fig. 3:**
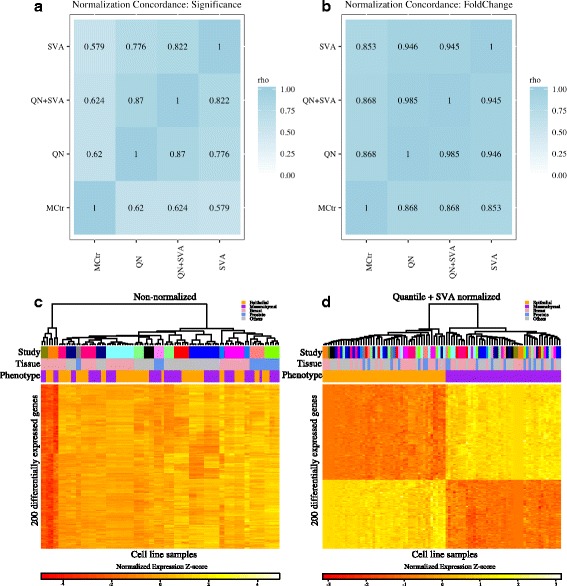
Consistency in differential expression signal across normalization methods. **a** Correlation heatmap showing concordance (Spearman rho) among ranks of differentially expressed genes using the four normalization methods (*n* = 7276). Genes were ranked by raw t-test *p*-values. **b** Correlation heatmap showing concordance (Spearman rho) among fold-change of differentially expressed genes using the four normalization methods (*n* = 7276). **c** Hierarchical Clustering of top 200 differentially expressed genes with uncorrected data shows strong clustering of samples by study rather than by phenotype. **d** Hierarchical Clustering of top 200 differentially expressed genes with QN + SVA (Quantile Normalized + SVA) corrected data clusters by epithelial and mesenchymal phenotype

Technical variability in the form of noise and batch-effects is inherent in gene expression data. We performed rigorous confounding factor correction to make gene expression comparisons between epithelial and mesenchymal samples that came from different studies, platforms, and cell lines. We used simple normalization methods including column standardized mean centered (MCtr) [35] and Quantile Normalization (QN) [33] and more rigorous methods that included Surrogate Variable Analysis (SVA) [34] and combination of QN followed by SVA (QN + SVA). With each normalization method (MCtr, QN, SVA, QN + SVA), we compared mean expression of epithelial and mesenchymal cell lines by a two-sample t-test for differential expression. We evaluated concordance among normalization methods to determine signal robustness – any individual method may be subject to false positives due to different patterns such as outliers, batch effects, etc. For this, we restricted our analysis to 7276 genes that were common across all studies. We used spearman correlation to test association between raw test statistics (*n* = 7276 genes) obtained from two-sample t-test from each of type of normalized data. Test-statistic distributions from individual normalization methods were significantly correlated with each other (*p*-value <2.2e-16, *n* = 7276). This indicates that signal produced by data normalized using a particular method is consistent with others (Fig. 3, Additional file 5: Figure S1, Additional file 6: Figure S2 and Additional file 7: Figure S3).

Next, to assess if normalization improved overall grouping of epithelial and mesenchymal phenotypes together, we clustered samples from each of the normalized datasets using hierarchical clustering (using all 7276 genes). Next, to evaluate this grouping we used the Baker Hubert Index (BH) with known phenotype vector as group assignments. Values of the BH index range from −1 to 1, with larger values indicating better grouping [48]. Table 3 shows that grouping of samples by phenotype (epithelial or mesenchymal) is considerably improved in normalized datasets in comparison to non-normalized data. QN + SVA performs the best, followed by SVA, MCtr and QN.

**Table 3:** Evaluation of sample grouping (with 7276 genes) using Baker Hubert index and phenotype information

	No normalization	Quantile Normalization (QN)	Surrogate Variable Analysis (SVA)	QN + SVA	Median Centered Column Scaled
Baker Hubert Index	0.0001	0.047	0.864	0.7995	0.0705

### Differential expression analyses reveal universal EMT genes across multiple carcinoma types

With every form of normalized data (MCtr, QN, SVA, QN + SVA), we determined differentially expressed genes between epithelial and mesenchymal cell phenotype by a two-sample t-test. A gene list ranked by raw *p*-values from the t-test was generated for each normalization method. Assuming equal likelihood of error in correction methods (Fig. 2), for each gene we assigned a differential rank that was the average of *p*-value ranks from all four normalization methods. This was used to generate a final integrated ranked gene list (Additional file 8: Table S2).

We defined a candidate universal EMT gene list by the top 200 genes from the integrated gene list (absolute fold change >1.2 and FDR < 0.005 in SVA, QN + SVA and MCtr normalized data) (Additional file 8: Table S2). These genes are representative of global differential EMT patterns independent of cell line origin and treatment modality.

Cancer cells recruit developmental pathways and processes to acquire migratory and invasive properties. To determine if the candidate gene list contained groups of genes working together and shared common biological functions we tested enrichment it’s enrichment for Hallmark genesets (MSigDB) defined and curated by the Broad Institute [49] using a right-tailed Fisher’s exact test. The most significantly enriched gene set was epithelial to mesenchymal transition (Odds ratio = 18.3575636, FDR = 4.92E-31). Among the other hallmark gene sets, we found increased representation (FDR < 10%) of several EMT related pathways including estrogen responsive genes (early and late), genes upregulated in response to low oxygen levels (hypoxia) and others [5, 50–57] (Table 4). We also found that specific estrogen responsive genes (early and late) were differentially expressed even when restricted just to the prostate cancer samples (Additional file 9: Figure S6) indicating this enrichment was not due exclusively to breast cancer cell lines in our combined analysis. When tested for GO biological processes, we found enrichment (FDR < 10%) for several developmental terms including epidermis development, anatomical structure morphogenesis and organ development (Additional file 10: Table S3). This further confirms that our analyses capture comprehensive signals in identifying changes in gene expression patterns across cancer types during EMT.

**Table 4:** Enriched MsigDB Hallmark genesets

Geneset	*p*-values	oddsratio	FDR	Genes in set
HALLMARK Epithelial mesenchymal transition	9.84E-33	18.3575636	4.92E-31	*CD59, CDH11, CDH2, COL1A1, COL1A2, COL4A2, COL5A1, COL6A3, CTGF, CYR61, DAB2, DPYSL3, EDIL3, EMP3, ENO2, FAP, FBN1, FBN2, FERMT2, GEM, GJA1, GREM1, LGALS1, LOX, MMP14, MMP2, PCOLCE, PCOLCE2, PLAUR, PLOD1, PMP22, POSTN, SERPINE1, SERPINE2, SLIT2, SPARC, SPOCK1, TGFB1, TIMP1, VCAN, VIM, WNT5A*
HALLMARK Estrogen response late	9.36E-06	4.332224532	0.00019652	*ALDH3A2, ASS1, CDH1, CELSR2, LLGL2, LSR, MAPK13, PLXNB1, RAPGEFL1, SCNN1A, SLC22A5, SLC27A2, ST14, TOB1, TRIM29*
HALLMARK Apical junction	1.18E-05	4.516129032	0.00019652	*AKT3, CDH1, CDH11, CLDN7, FBN1, GRB7, JAM3, JUP, MAPK13, MMP2, MPZL2, PVRL3, SLIT2, VCAN*
HALLMARK UV response dn	8.16E-05	4.23768997	0.001019448	*AKT3, COL1A1, COL1A2, CYR61, DAB2, FZD2, GJA1, HAS2, KCNMA1, MAP1B, PMP22, SERPINE1*
HALLMARK Estrogen response early	0.000247578	3.495078664	0.002475779	*AQP3, CELSR2, CLDN7, ELF3, GJA1, KRT15, PMAIP1, RAPGEFL1, SCNN1A, SLC22A5, SLC27A2, TOB1, WWC1*
HALLMARK Hypoxia	0.000436298	3.276838008	0.003635818	*AKAP12, CHST2, COL5A1, CTGF, CYR61, ENO2, ETS1, HMOX1, KDELR3, LOX, PLAUR, SERPINE1, SRPX*
HALLMARK Inflammatory response	0.000679488	3.786760716	0.004246802	*CD70, CHST2, EMP3, FZD5, HAS2, HRH1, MMP14, PLAUR, SERPINE1, TIMP1*
HALLMARK KRAS signaling up	0.00061698	3.554348835	0.004246802	*AKAP12, EPB41L3, ETS1, GFPT2, GNG11, JUP, MAP7, MPZL2, PLAUR, TMEM158, TRIB2*
HALLMARK Angiogenesis	0.003822541	7.2	0.02123634	*JAG2, POSTN, TIMP1, VCAN*
HALLMARK Complement	0.00451196	3.068992514	0.022559801	*CD59, COL4A2, CTSD, MMP14, PLAUR, SERPINE1, TIMP1, TIMP2, ZEB1*
HALLMARK Myogenesis	0.00594623	2.929880329	0.027028319	*COL1A1, COL4A2, COL6A3, ERBB3, MEF2C, NCAM1, PDLIM7, SPARC, TGFB1*
HALLMARK TGF beta signaling	0.010673511	4.097902098	0.044472964	*BCAR3, CDH1, SERPINE1, SMURF2, TGFB1*

Among genes on our candidate gene list, we found known epithelial- and mesenchymal-specific genes such as E-cadherin (*CDH1*), Zinc Finger E-Box Binding Homeobox 1 (*ZEB1*), Vimentin (*VIM*), Transforming Growth Factor, Beta 1 (*TGFB1*), Tissue Inhibitor Of Metalloproteinase 1 (*TIMP1*) [5, 58], N-cadherin (*CDH2*) (Table 5). We also observed enrichment of collagen genes that are known to be associated with cell adhesion and migration amongst DE genes (Fisher’s exact *p*-value 1.124e-05) [5]. In addition, we also found known EMT related transcription factors such as *ZEB1*, *ETS1* and *LSR* in our candidate gene list.

**Table 5:** Rank of known epithelial and mesenchymal specific genes and DE genes found in Hallmark Epithelial to mesenchymal transition [73]

Gene Symbol	Order in average rank	Gene Symbol	Order in average rank	Gene Symbol	Order in average rank
*EMP3*	3	*PCOLCE2*	45	VIM*	144
*VCAN*	4	*FAP*	54	*FERMT2*	147
*GEM*	6	*CDH11*	73	*POSTN*	150
CDH2*	11	TGFB1*	81	*FBN2*	155
ZEB1*	12	*SPARC*	84	*GJA1*	159
*SPOCK1*	13	*CYR61*	90	*SERPINE1*	161
*COL4A2*	14	*WNT5A*	95	*DAB2*	168
*FBN1*	15	*CD59*	98	*COL1A1*	171
*PMP22*	21	*GREM1*	106	*MMP2*	174
*COL5A1*	22	*PLAUR*	108	*PCOLCE*	181
CDH1*	24	*CTGF*	118	*ENO2*	187
*SLIT2*	33	*COL1A2*	120	*LGALS1*	191
*EDIL3*	36	*PLOD1*	124	*SERPINE2*	162
*DPYSL3*	42	*MMP14*	127		
*COL6A3*	43	*LOX*	129		

*commonly used EMT marker genes

We also compared our list of genes to the core EMT gene signature described by Groger et al. [27]. We found 43 common genes from their study (Additional file 11: Table S4). These included genes such as *CDH1, CDH2, VIM, LSR* and some collagen genes. Several known EMT genes such as *TGFB*, *TIMP1*, *ETS1* that were found in universal EMT genes were missing from their list. Some other genes such as *S100A14*, *DPYSL3* and *C1orf116* (Additional file 12: Figure S4 and Additional file 13: Figure S5) that we validate as differential EMT genes in our study, were also not found in their core gene list.

### Candidate gene list identified genes previously unknown in prostate cancer EMT

In addition to genes well established in the process of EMT, we also identified genes that had only been described in EMT in a subset of cancer types, including two epithelial specific genes, lipolysis stimulated lipoprotein receptor (*LSR*) and S100 calcium binding protein A14 (*S100A14*), and one mesenchymal specific gene, dihydropyrimidinase-like 3 (*DPYSL3*). Previous studies have investigated role of *LSR* in breast cancer EMT [59], and *S100A14* has been examined in pancreatic and cervical cancer [60, 61]. Previous studies have indicated involvement of *DPYSL3* in malignant pancreatic and gastric tumors [62, 63].

**Fig. 4:**
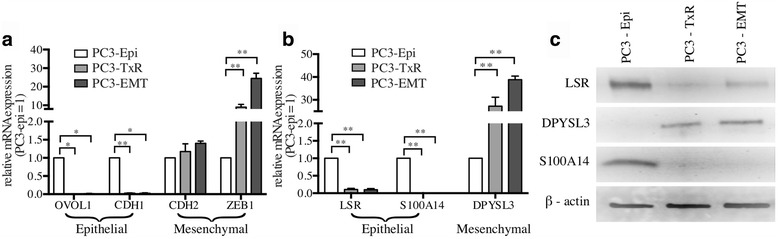
Expression of EMT associated genes in prostate cancer EMT. **a** qPCR: mRNA expression of known epithelial and mesenchymal specific genes in PC3 prostate cancer EMT model cell line. **b** qPCR: mRNA expression of epithelial and mesenchymal specific genes in PC3 prostate cancer cell lines previously unknown in prostate cancer EMT. * *P* < 0.05; ** *P* < 0.005; *** *P* < 0.0005. **c** Immunoblot: Protein expression of epithelial and mesenchymal specific genes in PC3 prostate cancer cell lines previously unknown in prostate cancer EMT (LSR, DPYSL3, S100A14, C1orf116, and β-actin were all probed on the same blot, so the β-actin loading control is appropriate for both Fig. 4c (LSR, DPYSL2, S100A14) and Fig. 5b (C1orf116). Data were separated into two figures for clarity)

We validated the expression of these genes in an in vitro model of prostate cancer EMT. mRNA and protein expression levels of these genes were determined in one epithelial and two mesenchymal prostate cancer cell line PC3 derivatives. PC3-Epi is an expansion of a highly epithelial clone from the parental PC3 population. The mesenchymal derivatives were generated from PC3 cells by M2 macrophage co-cultures (PC3-EMT) and Taxol treatment and subsequent resistance (PC3-TxR) [20, 64]. RT-qPCR of canonical epithelial and mesenchymal genes, *OVOL1*, *OVOL2*, *CDH1*, *ZEB1*, and *CDH2*, confirmed the appropriate phenotypic states for these cells lines (Fig. 4a). Elevated levels of *S100A14* mRNA was observed in PC3-Epi compared to mesenchymal PC3-EMT and PC3-TxR. Similarly, mRNA expression of epithelial gene *LSR* was found to be higher in PC3-Epi than in its mesenchymal counterparts, PC3-EMT and PC3-TxR (Fig. 4b).

Conversely, the mesenchymal gene *DPYSL3* was extremely upregulated in PC3-EMT and PC3-TxR than in PC3-Epi (Fig. 4b). These results were supported by western blot analysis, which demonstrated protein levels mirrored the mRNA expression (Fig. 4c).

### *C1orf116* was discovered to be a novel EMT regulator

**Fig. 5:**
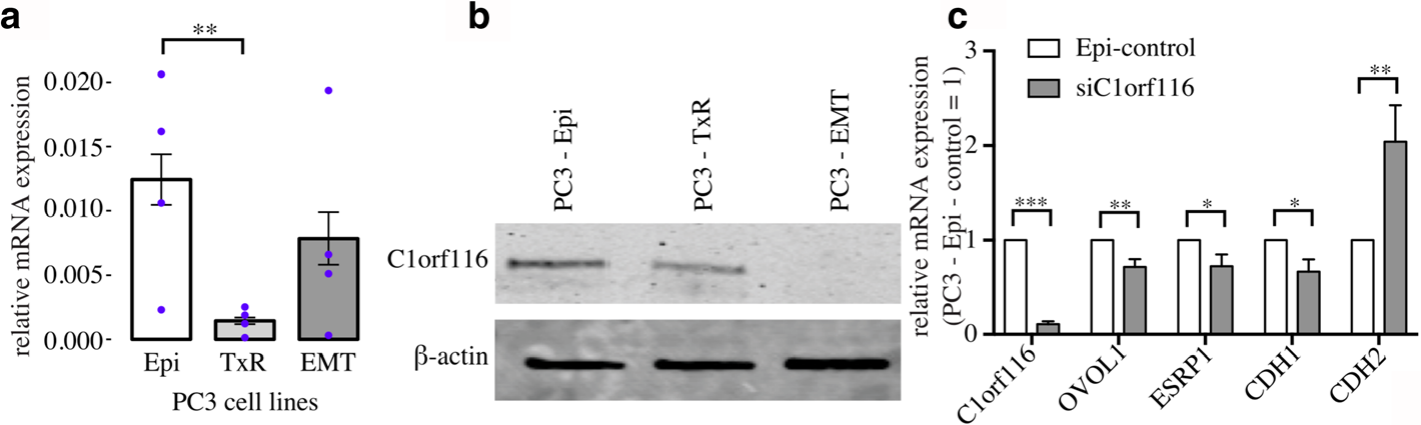
*C1orf116*: a novel EMT regulator. **a** qPCR: mRNA expression of *C1orf116* in EMT model prostate cancer cell lines PC3-Epi, PC3-EMT and PC3-TxR * *P* < 0.1; ** *P* < 0.05; *** *P* < 0.005. **b** Immunoblot: Protein expression of C1orf116 in EMT model prostate cancer cell lines PC3-Epi, PC3-EMT and PC3-TxR (LSR, DPYSL3, S100A14, C1orf116, and β-actin were all probed on the same blot, so the β-actin loading control is appropriate for both Fig. 4c (LSR, DPYSL2, S100A14) and Fig. 5b (C1orf116). Data were separated into two figures for clarity). **c** qPCR: mRNA expression of *C1orf116* and other known epithelial (*OVOL1, ESRP1 and CDH1*) and mesenchymal (*CDH2*) gene in PC3-Epi cells transfected with *C1orf116*-siRNA relative to empty vector control * *P* < 0.1; ** *P* < 0.05; *** *P* < 0.005

Our candidate gene list also contained genes that have not been previously described as related to the EMT process in any cancer type or in any physiologic process. One of these novel candidate EMT genes, *C1orf116* (also known as *SARG*), is a poorly characterized gene with only one PubMed listed publication [65]. We first validated our finding from microarray data using the PC3 in vitro model of EMT and found increased mRNA expression in PC3-Epi cells compared to PC3-emt (1.3 fold) and PC3-TxR (8.8 fold). These results were supported by elevated protein expression of C1orf116 in PC3-epi cells (Fig. 5a-b).

**Fig. 6:**
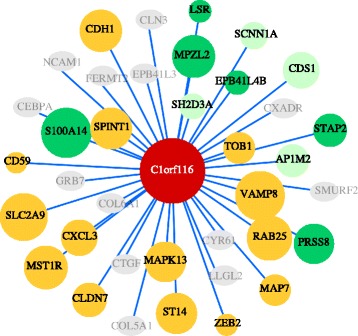
*C1orf116* associated genes in weighted gene correlation network module. This correlation network shows association of *C1orf116* module genes obtained from WGCNA. Node size is a function of correlation with *C1orf116* expression. Yellow nodes represent genes that have been previously studied in multiple (greater than 3) cancer types. Bright green nodes are the genes that have been studied in 3 or less cancer types. Light green nodes are genes that have not been specifically studied in cancer. Gray nodes were genes that were not significantly associated with expression of *C1orf116*

Increased expression *C1orf116* in epithelial cells confirmed of it as an epithelial marker gene. We applied gene network analysis [37], that revealed weighted coexpression gene modules (groups of co-expressed genes) and showed that *C1orf116* clustered with other epithelial genes including *CDH1, LSR, S100A14* and others (Additional file 14: Table S5, Fig. 6). *LSR* and *S100A14* were among the known-unknown genes whose expression was validated in PC3 cell lines. This confirmed its association with other epithelial genes universal across other disease types. Through manual literature search, we identified that a subset of the *C1orf116* module gene list have been shown to be associated with multiple cancer types. Among other genes in the modules, *SH2D3A, AP1M2, CDS1* and *SCNN1A* haven’t been previously studied in cancer biology. This shows that in addition to being a novel EMT regulator in prostate cancer, *C1orf116* could have broad effects across multiple cancer types.

**Fig. 7:**
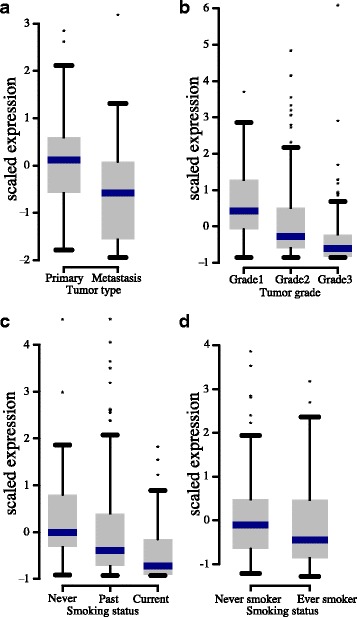
*C1orf116* expression in cancer patient data. **a** Decreased expression of C1orf116 is seen in metastatic tumor type compared to primary prostate cancer (Taylor dataset); unadjusted *P* = 0.0340, Bonferroni adjusted *P* = 0.51. **b** Expression of C1orf116 decreases in high grade lung cancer (Director’s challenge dataset); Bonferroni adjusted *P* < 0.0005. **c** *C1orf116* is downregulated in lung cancer patients with increased smoking habits (Director’s challenge dataset); unadjusted *P* < 0.01, Bonferroni adjusted *P* < 0.1. **d** *C1orf116* is downregulated in lung cancer patients with smoking habits in comparison to non-smokers (Okayama dataset); unadjusted *P* = 0.0586, Bonferroni corrected *P* = 0.879

Next, we interrogated the possible role of *C1orf116* in in vivo malignant progression. For this, we identified gene expression studies with at least 150 patients that also had information on tumor grade and expression data for *C1orf116* and were able to find breast, prostate, colorectal and lung cohorts (Additional file 4: Figure S7). We found that *C1orf116* expression is decreased in metastatic lesions compared to localized tumors in prostate cancer patients (Fig. 7a) [46]. Likewise, *C1orf116* expression decreased with increasing cancer grade in patients with lung cancer (Fig. 7b) [44]. Studies have shown that lung cancer patients with history of smoking tobacco/cigarette exhibit lower expression levels of E-cadherin and higher levels of mesenchymal markers such as vimentin [66, 67]. Previous studies have also indicated that cigarette smoking can induce EMT in non-small cell lung cancer [68]. Analogous to these findings, we observed reduced expression of *C1orf116* among lung cancer patients with smoking habits (Fig. 7c-d) [44, 45]. In some breast cancer datasets expression of *C1orf116* increased with increasing cancer grade (Additional file 3: Table S7 and Additional file 4: Figure S7). This suggested that in addition to expression changes in in vitro cell line models, changes in *C1orf116* expression could potentially have a functional role in clinically-important disease progression in cancer patients.

To test the role of *C1orf116* as a driver of an epithelial phenotype, we used siRNA-mediated knockdown of the gene in PC3-Epi cells. We found that siRNA-mediated knockdown of *C1orf116* expression resulted in decreased expression of epithelial markers *OVOL1*, *ESRP1*, and *CDH1*, and increased expression of mesenchymal marker *CDH2* (Fig. 5c). This suggests that *C1orf116* plays a functional role in maintaining epithelial phenotype. Significant upregulation of mesenchymal genes in response to *C1orf116* knockdown indicates it as a novel regulator of EMT.

## Discussion

EMT may be an early step in cancer metastasis and has been associated with chemoresistance and disease progression [69, 70]. Though EMT is common among all solid tumor types and is essential in early development, common drivers of EMT across multiple cancer types have not been described. Several studies have investigated EMT in cell lines from within a single disease type. Although most studies have been confined to very small sample size. To address this, we systematically integrate multiple EMT studies to increase power and identify novel drivers of EMT universal to all cancer types.

A significant challenge in multi-study analysis comes from various sources of heterogeneity arising from study specific technical and biological variation. Biological variation interferes with analyses, especially when it is not the signal of interest. We employed two strategies to address various sources of heterogeneity and noise. First, we chose stringent normalization methods that have been shown to reduce the influence of such heterogeneity (SVA, quantile normalization, and scaled median centering). We recognize that these methods may have their failure modes and limitations. Therefore, we defined our final differentially expressed gene list from consensus ranking across all four normalization schemes. Thus even if a single method introduced an error or failed to account for a particular effect, the final gene list may be more robust than results from any individual method. However, technical variation and experimental heterogeneity may still influence the results of our analysis, as no method has been shown to fully remove such effects from expression data. Therefore, experimental validation and comparison with external functional annotation were important.

Integrating across multiple studies did improve power and helped us detect novel genes that showed consistent effect across multiple studies, which could be concealed in a single study. We found three groups of genes in the EMT differentially expressed list: a) known EMT genes (e.g. *CDH1*, *ZEB1*, *TGFB*, *CDH2*, *VIM*, *TIMP1*), b) EMT genes previously unknown in prostate cancer (*LSR*, *S100A14*, *DPYSL3*) and c) novel EMT genes (including *C1orf116*).

We confirmed our discovery of unknown EMT genes in prostate cancer by testing expression of *LSR*, *S100A14*, *and DPYSL3* in a PC3 prostate cancer cell line model of EMT. Previous studies have shown that LSR suppresses EMT phenotype in claudin-low breast cancer cell lines [59]. *S100A14* has been studied in breast cancer progression and is showed to be involved in EMT in human cervical and pancreatic cancer cells [60, 61, 71]. *DPYSL3* is associated with malignant gastric and pancreatic tumors [62, 63]. Moreover studies suggest that mRNA expression of *DPYSL3* is positively correlated with Vascular Endothelial Growth Factor (*VEGF*), a gene thought to be involved in EMT [72]. This data indicates that our method bridged EMT cancer biology across different disease types and captures global expression patterns in EMT (Additionale file 12: Figure S4A-C).

We confirmed discovery of *C1orf116* as epithelial specific gene by testing its expression in PC3 in vitro model of EMT. siRNA knockdown of *C1orf116* in PC3 epithelial cell lines showed loss of epithelial markers and gain of mesenchymal markers thereby confirming its functional role as a negative driver of EMT. Clinical data from breast, prostate cancer and lung cancer patients also suggested that changes in expression of *C1orf116* could have functional implications in disease progression.

Altogether, through this study we have found genes whose effects are represented by multiple cancer types (breast, prostate, liver, colon, esophagus and retinal pigment). We have also validated expression of some genes in an in vitro prostate cancer cell line model and potential relevance in vivo data from three tissues, including one (lung) that was not represented among our cell line data. However, these effects might not necessarily be extrapolated for cancer types not included in this study. As data become available for other tissues and cancers, further analysis can be performed.

## Conclusions

Using multi-study integration approach, we identified consensus ranked universal EMT genes. This gene list comprised of a) known EMT genes that included *CDH1*, *ZEB1* and *CDH2* b) genes studied in a subset of carcinomas, unknown in prostate cancer: *LSR*, *S100A14* and *DPYSL3* and c) novel unknown EMT and cancer genes such as *C1orf116*. siRNA experiments indicate it to be a potential novel regulator of EMT. Patient gene expression data shows that reduced expression of *C1orf116* is associated with poor prognosis in lung and prostate cancer (unadjusted Wilcoxon rank sum *p*-value <0.05). In conclusion, our approach of statistical analysis and functional validation identified universal EMT genes and candidate global regulatory genes, thereby both extending current knowledge of EMT and showed preliminary evidence of disease progression in cancer.

## Additional files

Additional file 1: Table S1. Dataset information – Extended table. (XLSX 33 kb)

Additional file 2: Table S6. List of antibodies used in immunoblot. (XLSX 36 kb)

Additional file 3: Table S7. Association of *C1orf116* expression in lung and prostate cancer patients. (XLSX 9 kb)

Additional file 4: Figure S7. *C1orf116* expression in clinical patient data from breast and colorectal cancer. (PDF 25 kb)

Additional file 5: Figure S1. Hierarchical Clustering of top 200 differentially expressed genes with data corrected by quantile normalization. (PDF 429 kb)

Additional file 6: Figure S2. Hierarchical Clustering of top 200 differentially expressed genes with data corrected by SVA (Surrogate Variable Analysis) normalization. (PDF 122 kb)

Additional file 7: Figure S3. Hierarchical Clustering of top 200 differentially expressed genes with data corrected by MCtr (Median Centered Column Scaled) normalization. (PDF 122 kb)

Additional file 8: Table S2. Gene ranks – fold change and significance based. (XLSX 1503 kb)

Additional file 9: Figure S6. Expression of Estrogen responsive genes - (A) early and (B) late in prostate cancer cell line samples from integrated data. (PDF 115 kb)

Additional file 10: Table S3. Gene set enrichment with GO Biological Processes term. (XLSX 36 kb)

Additional file 11: Table S4. Common genes with Groger et al. study and 200 DE genes. (XLSX 32 kb)

Additional file 12: Figure S4. Expression of EMT genes previously unknown in prostate cancer in integrated cell lines data. Expression of *LSR* (A), *S100A14* (B) and *DPYSL3* (C) in breast, prostate and others (retinal pigment, liver, colon and esophageal) cancer cell lines from QN + SVA normalized integrated data. (PDF 97 kb)

Additional file 13: Figure S5. Expression of *C1orf116* in breast, prostate and others (retinal pigment, liver, colon and esophageal) cancer cell lines from integrated data. (PDF 45 kb)

Additional file 14: Table S5. C1orf116 module genes obtained from Weighted Gene Co-expression analysis. (XLSX 42 kb)

## Abbreviations

EMT: Epithelial to mesenchymal transition
MCtr: Median Centered Column Scaled
MET: Mesenchymal to epithelial transition
QN: Quantile Normalization
RT-qPCR: Reverse transcription quantitative polymerase chain reaction
SVA: Surrogate Variable Analysis
WGCNA: Weighted Gene Coexpression Network Analysis
