# Supplementary material for: Identifying global expression patterns and key regulators in epithelial to mesenchymal transition through multi-study integration
Source: BMC Cancer. 2017 Jun 26;17:447. doi: 10.1186/s12885-017-3413-3 (PMC5485747; doi:10.1186/s12885-017-3413-3)
Supplement: Supplementary file 4 — C1orf116 expression in clinical patient data from breast and colorectal cancer. (PDF 25 kb) [file 12885_2017_3413_MOESM4_ESM.pdf]

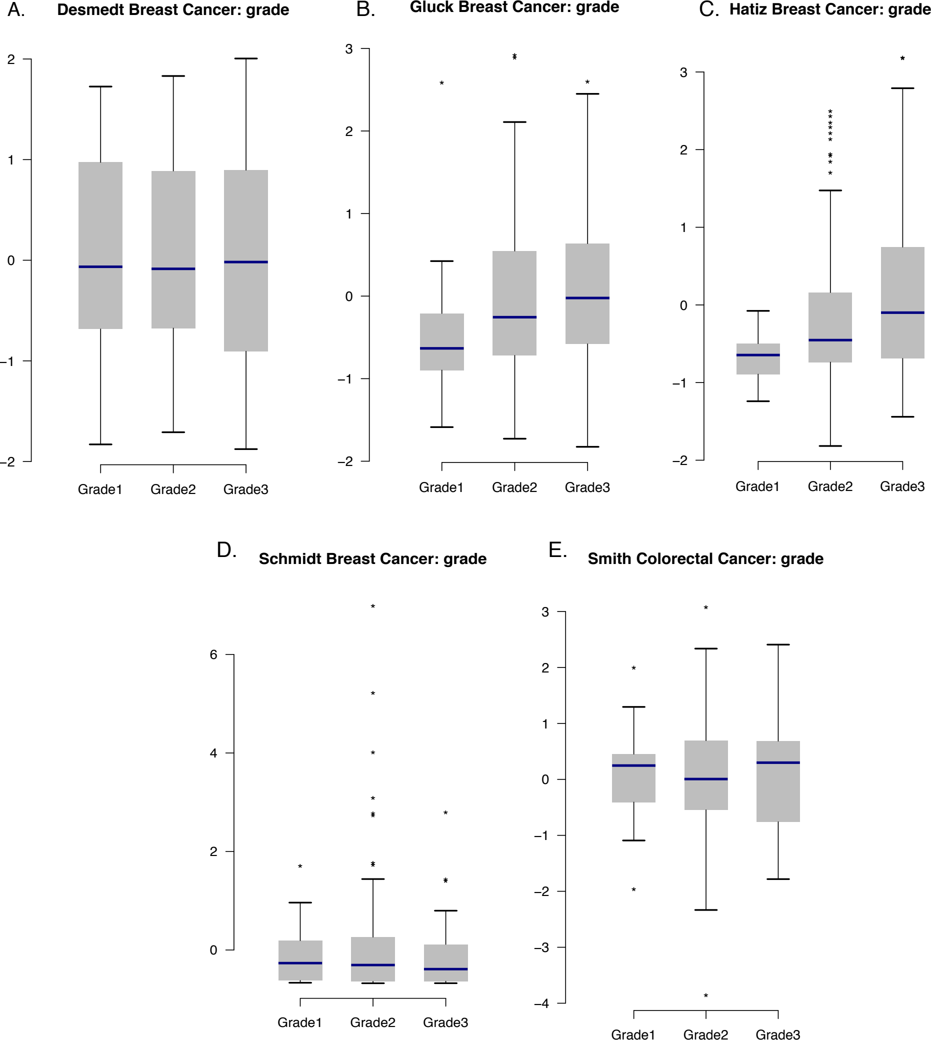
A.
Desmedt Breast Cancer: grade
2
1
0
−1
−2
Grade1
Grade2
Grade3
B.
Gluck Breast Cancer: grade
3
2
1
0
−1
−2
Grade1
Grade2
Grade3
C.
Hatiz Breast Cancer: grade
3
2
1
0
−1
−2
Grade1
Grade2
Grade3
D.
Schmidt Breast Cancer: grade
6
4
2
0
Grade1
Grade2
Grade3
E.
Smith Colorectal Cancer: grade
3
2
1
0
−1
−2
−3
−4
Grade1
Grade2
Grade3
