# Supplementary figures and images for: Identifying global expression patterns and key regulators in epithelial to mesenchymal transition through multi-study integration
Source: BMC Cancer. 2017 Jun 26;17:447. doi: 10.1186/s12885-017-3413-3 (PMC5485747; doi:10.1186/s12885-017-3413-3)

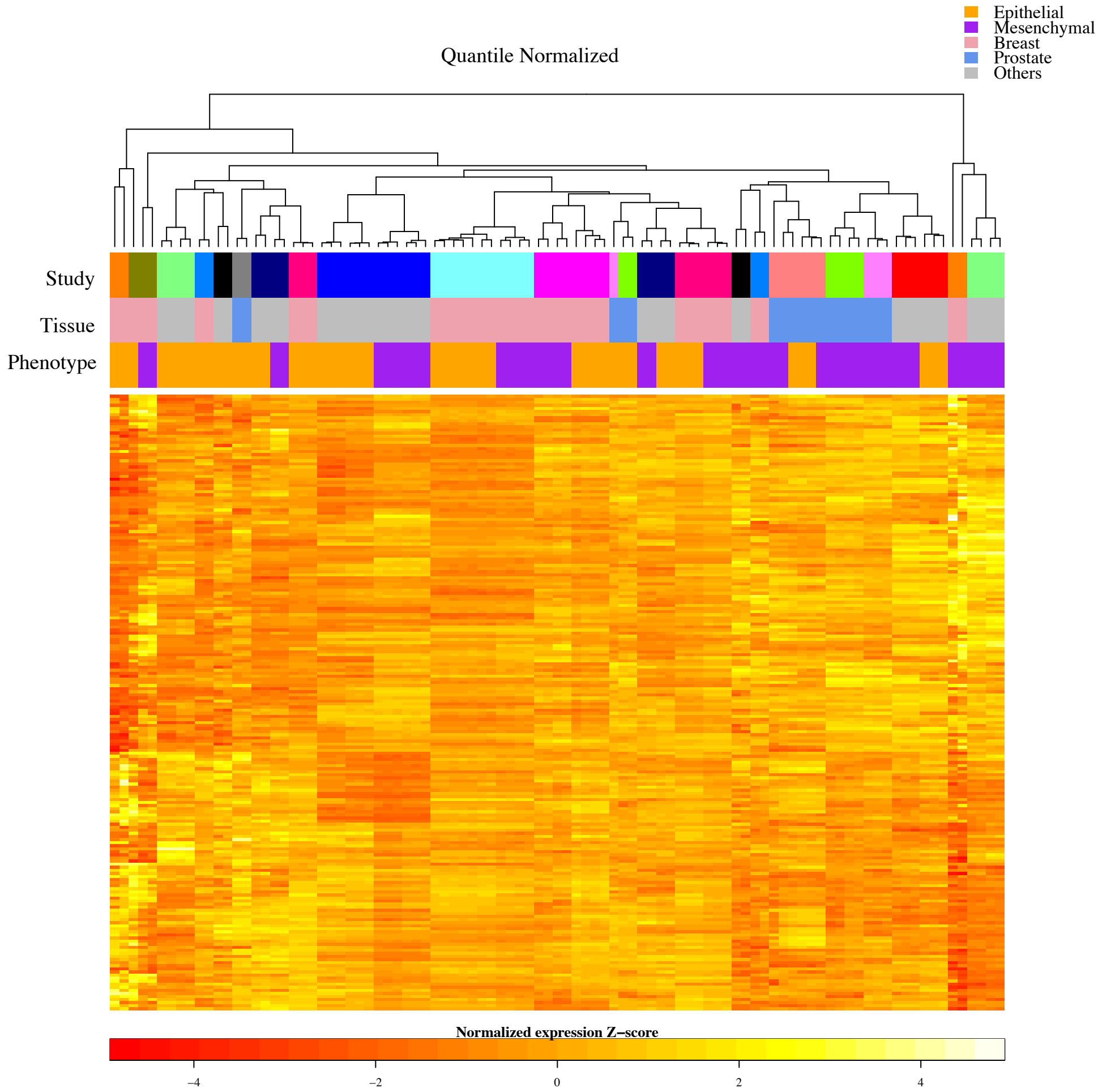

Supplement: Supplementary file 5 — Hierarchical Clustering of top 200 differentially expressed genes with data corrected by quantile normalization. (PDF 429 kb) [file 12885_2017_3413_MOESM5_ESM.pdf]

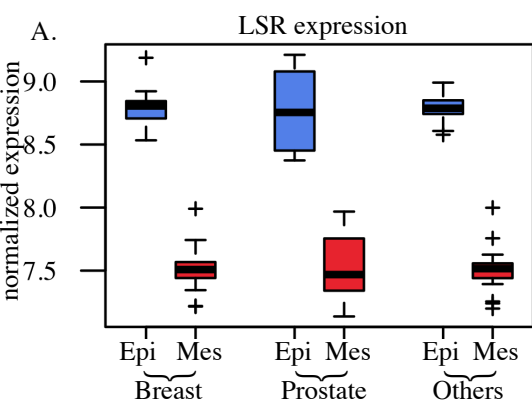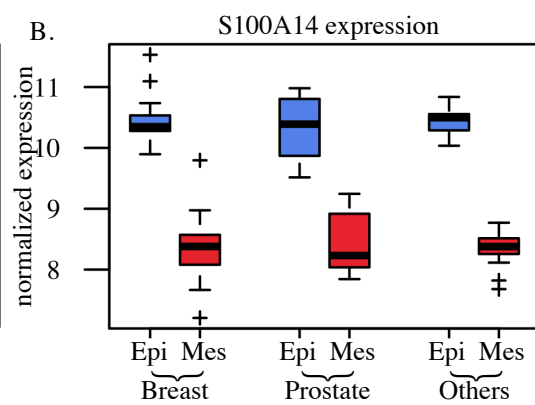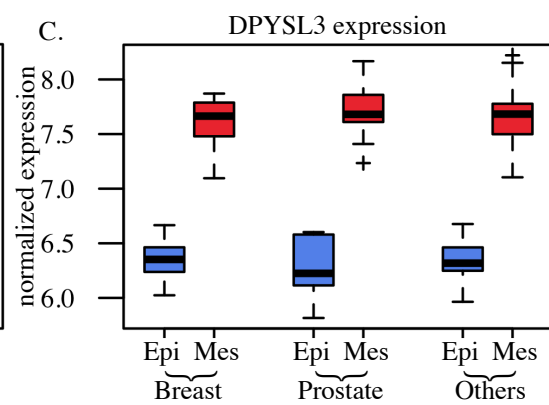

Supplement: Supplementary file 12 — Expression of EMT genes previously unknown in prostate cancer in integrated cell lines data. Expression of LSR (A), S100A14 (B) and DPYSL3 (C) in breast, prostate and others (retinal pigment, liver, colon and esophageal) cancer cell lines from QN + SVA normalized integrated data. (PDF 97 kb) [file 12885_2017_3413_MOESM12_ESM.pdf]

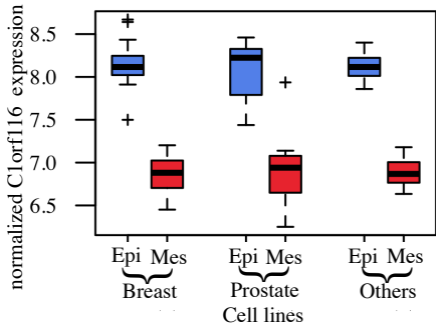

Supplement: Supplementary file 13 — Expression of C1orf116 in breast, prostate and others (retinal pigment, liver, colon and esophageal) cancer cell lines from integrated data. (PDF 45 kb) [file 12885_2017_3413_MOESM13_ESM.pdf]
