# Supplementary material for: Identifying global expression patterns and key regulators in epithelial to mesenchymal transition through multi-study integration
Source: BMC Cancer. 2017 Jun 26;17:447. doi: 10.1186/s12885-017-3413-3 (PMC5485747; doi:10.1186/s12885-017-3413-3)
Supplement: Supplementary file 6 — Hierarchical Clustering of top 200 differentially expressed genes with data corrected by SVA (Surrogate Variable Analysis) normalization. (PDF 122 kb) [file 12885_2017_3413_MOESM6_ESM.pdf]

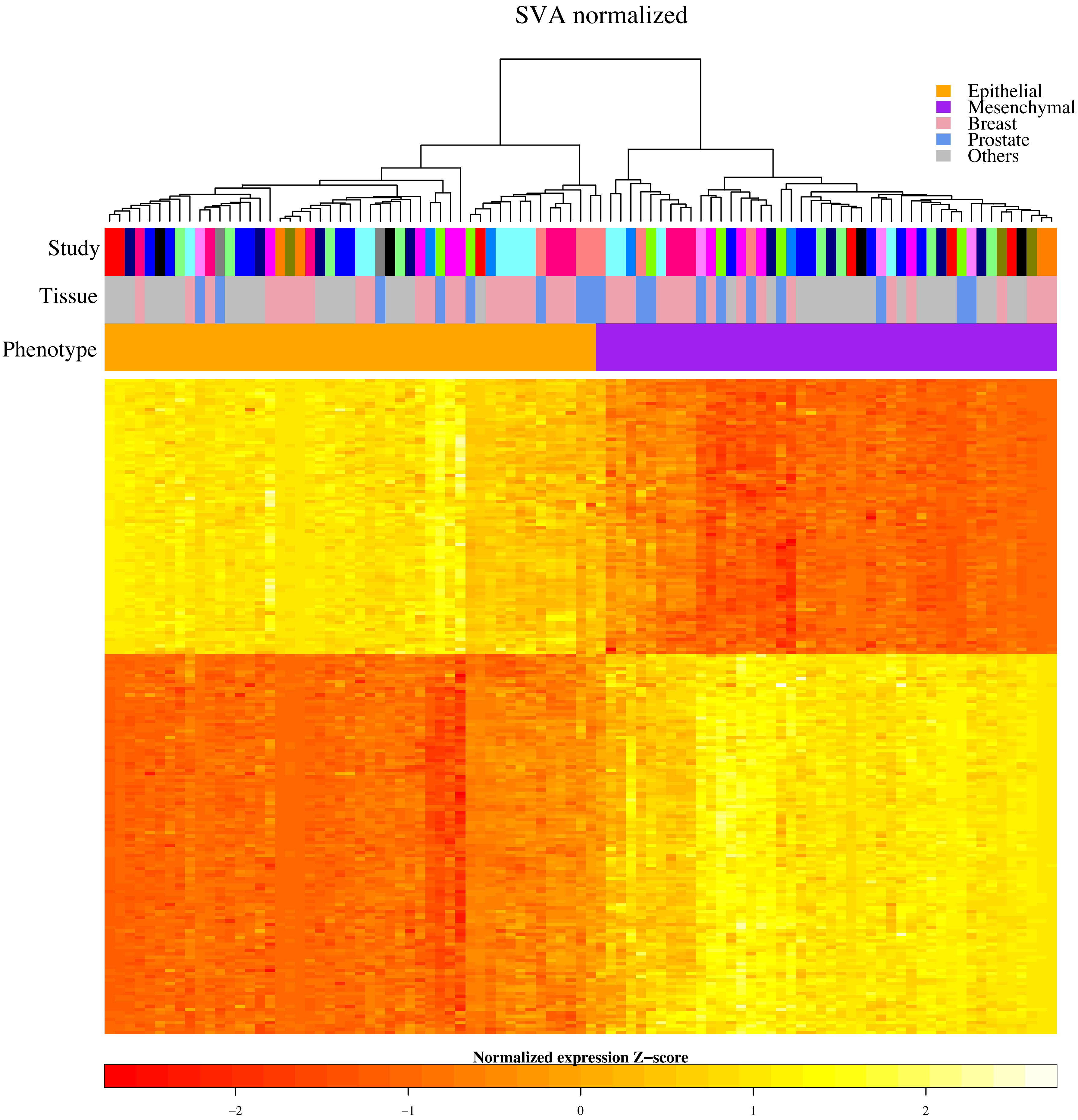
SVA normalized
Epithelial
Mesenchymal
Breast
Prostate
Others
Study
Tissue
Phenotype
Normalized expression Z−score
−2
−1
0
1
2
