# Supplementary material for: Identifying global expression patterns and key regulators in epithelial to mesenchymal transition through multi-study integration
Source: BMC Cancer. 2017 Jun 26;17:447. doi: 10.1186/s12885-017-3413-3 (PMC5485747; doi:10.1186/s12885-017-3413-3)
Supplement: Supplementary file 9 — Expression of Estrogen responsive genes - (A) early and (B) late in prostate cancer cell line samples from integrated data. (PDF 115 kb) [file 12885_2017_3413_MOESM9_ESM.pdf]

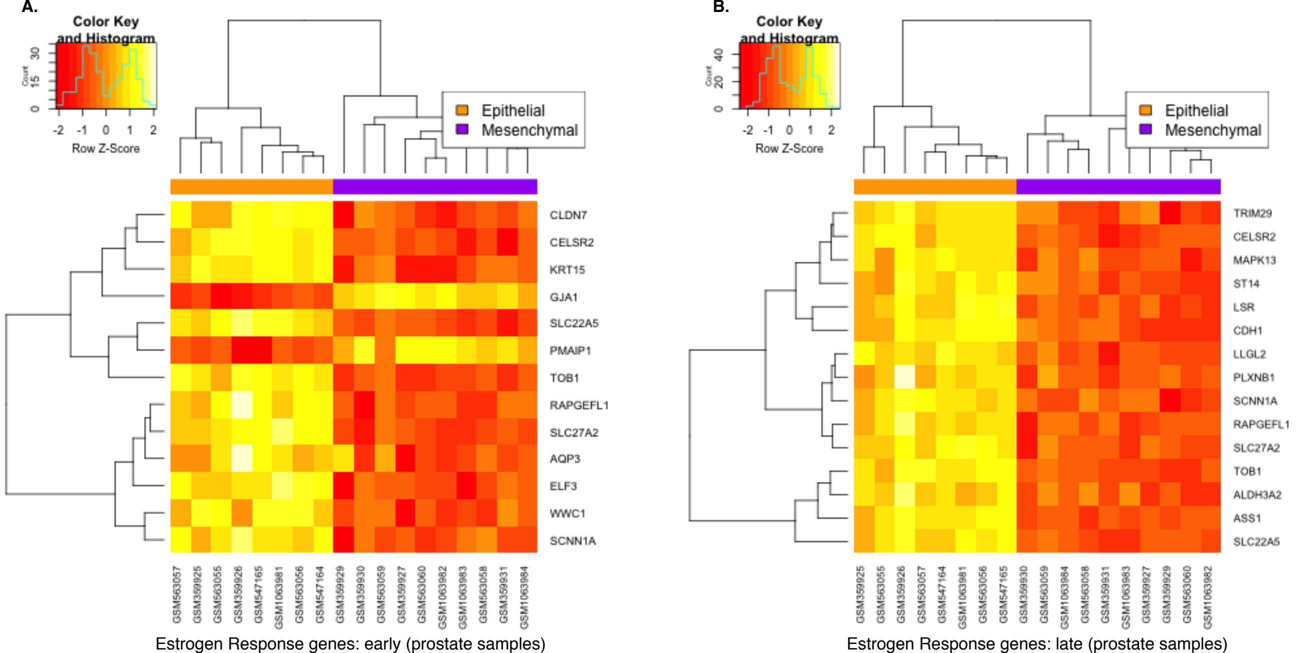

A.
Color Key and Histogram
Count
Row Z-Score
Epithelial
Mesenchymal
CLDN7
CELSR2
KRT15
GJA1
SLC22A5
PMAIP1
TOB1
RAPGEFL1
SLC27A2
AQP3
ELF3
WWC1
SCNN1A
GSM563057
GSM359925
GSM563055
GSM359926
GSM547165
GSM1063981
GSM563056
GSM547164
GSM359929
GSM359930
GSM563059
GSM359927
GSM563060
GSM1063982
GSM1063983
GSM563058
GSM359931
GSM1063984
Estrogen Response genes: early (prostate samples)
B.
Color Key and Histogram
Count
Row Z-Score
Epithelial
Mesenchymal
TRIM29
CELSR2
MAPK13
ST14
LSR
CDH1
LLGL2
PLXNB1
SCNN1A
RAPGEFL1
SLC27A2
TOB1
ALDH3A2
ASS1
SLC22A5
GSM359925
GSM563055
GSM359926
GSM563057
GSM547164
GSM1063981
GSM563056
GSM547165
GSM359930
GSM563059
GSM1063984
GSM563058
GSM359931
GSM1063983
GSM359927
GSM359929
GSM563060
GSM1063982
Estrogen Response genes: late (prostate samples)
